# Beyond Individual Fault: A Context-Sensitive Framework for Assessing Medical Liability in Obstetrics and Gynaecology

**DOI:** 10.3390/healthcare14203531

**Published:** 2026-10-20

**Authors:** Larisa Pătru, Maria Cristina Comănescu, Elena Cristina Andrei, Daniela-Maria Micu, Răzvan Grigoraș Capitanescu, Ciprian Laurențiu Pătru

**Affiliations:** 1 Department 9, Faculty of Medicine, University of Medicine and Pharmacy of Craiova, 200349 Craiova, Romania; larisa.patru@umfcv.ro; 2 Department of Anatomy, Faculty of Medicine, University of Medicine and Pharmacy of Craiova, 200349 Craiova, Romania; maria.comanescu@umfcv.ro; 3 Department of Histology, Faculty of Dentistry, University of Medicine and Pharmacy of Craiova, 200349 Craiova, Romania; elena.andrei@umfcv.ro; 4 Research Centre for Microscopic Morphology and Immunology, University of Medicine and Pharmacy of Craiova, 200349 Craiova, Romania; 5 Dental Medicine Research Center, University of Medicine and Pharmacy of Craiova, 200349 Craiova, Romania; 6 Department of Foreign Languages, University of Medicine and Pharmacy of Craiova, 200349 Craiova, Romania; daniela.micu@umfcv.ro; 7 Department 8 Mother and Child, University of Medicine and Pharmacy of Craiova, 200349 Craiova, Romania; ciprian.patru@umfcv.ro

**Keywords:** medical liability, obstetrics and gynaecology, medical malpractice, clinical judgement, standard of care, causation, patient autonomy, patient safety, healthcare systems

## Abstract

Background/Objectives: Previous obstetric and gynaecological malpractice reviews identify recurring concerns but do not specify a sequence for examining conduct before deviation, causation and attribution. We developed a context-sensitive conceptual framework. Methods: A structured critical narrative review of PubMed/MEDLINE, Scopus and Web of Science Core Collection was combined with analysis of Romanian legal and professional sources. The broad searches yielded 1785 database records before deduplication. The team’s legal specialist manually screened the overlapping thematic searches 2A–2D using her subject expertise; duplicates were removed and 223 unique publications were retained. The 347 records reported for thematic search 2A alone are not the input denominator for that corpus. She extracted the information for 40 selected anchor publications, mapping them to eight broad themes. The thirteen-domain Context-Sensitive Liability Assessment Framework for Obstetrics and Gynaecology (CSLAF-OG) was illustrated using three principal Romanian judicial cases and one additional case. Results: The framework orders context, professional standards, autonomy, communication, documentation and organisational conditions before harm, deviation, causation and attribution. Conclusions: CSLAF-OG is a conceptual aid for retrospective analysis, not a validated instrument or legal test. Independent evaluation remains necessary.

## 1. Introduction

Obstetric and gynaecological care occupies a distinctive position in contemporary medicine because clinical decisions are frequently made under conditions of uncertainty, time pressure, competing risks, and potentially severe maternal or neonatal consequences. At the same time, these decisions engage patient autonomy, informed consent, reproductive choice, professional liability and institutional accountability. The resulting medico-legal environment is therefore shaped not only by the technical quality of individual clinical acts, but also by how information is interpreted, how reasonable alternatives are weighed, how professional standards are applied, how decisions are communicated and documented, and how organisational conditions affect the delivery of care.

Medical liability in obstetrics and gynaecology is commonly discussed through professional negligence, standard of care, causation, informed consent, diagnostic or treatment delay, documentation, and litigation exposure [1,2,3,4,5,6,7,8,9,10,11,12]. These domains recur across prenatal diagnosis, intrapartum monitoring, operative delivery, postoperative care, communication, and escalation pathways. Their recurrence does not, however, justify reasoning from a severe adverse outcome directly to professional fault. Liability assessment requires separate consideration of the clinical event, the existence of any breach of the applicable professional standard of care, the reasonableness of the clinician’s conduct at the relevant time, the causal contribution of that breach to the harm, and the final attribution of responsibility. This separation is particularly important in obstetric practice, where several defensible alternatives may coexist, and retrospective knowledge of outcome can alter perceptions of what should have been apparent earlier. Qualitative evidence shows that obstetricians negotiate professional standards, experience, patient preferences, institutional policy, and malpractice concerns rather than applying any single source of authority in isolation [9,13,14,15]. Outcome and hindsight bias further complicate retrospective evaluation [16,17].

Diagnostic failure illustrates the same problem. In a medico-legal analysis of 73 claims concerning obstetric ultrasonography, all cases involved undiagnosed congenital anomalies; however, only four cases were considered by the Council of Forensic Medicine to constitute malpractice. Diagnostic limitations, examiner competence, and compliance with the applicable professional standards were relevant to the assessment [18]. More recent literature similarly emphasises the technical limits of antenatal ultrasound and the need to distinguish non-detection from unreasonable care [19,20,21].

Existing reviews have already described many principal sources of malpractice exposure in obstetrics and gynaecology. Most notably, Toma-Tumbar et al. evaluated 25 articles and discussed stakeholders, standards of care, diagnostic errors, complications, patient counselling, surgery, informed consent, documentation, and legal implications [1]. The present study therefore does not claim novelty from identifying these factors. Its incremental contribution is methodological: it organises established domains into an explicit ex ante sequence, separates outcome, deviation, causation, and attribution, and explores that sequence against Romanian judicial material. Table 1 makes this distinction explicit.

The residual gap concerns how contextual and systemic influences can be organised into an explicit obstetrics- and gynaecology-specific analytical sequence. The sequence begins with contemporaneous information and realistic alternatives, incorporates professional expectations, autonomy, communication, documentation, capability and organisational conditions, and then addresses harm, deviation, causation and attribution.

To address this gap, the present study develops the Context-Sensitive Liability Assessment Framework for Obstetrics and Gynaecology (CSLAF-OG). The framework treats retrospective reconstruction as the starting point and liability attribution as the end point. Its novelty lies in integration and sequencing rather than in presenting any individual domain as new.

The central research question is: How can medical liability in obstetric and gynaecological care be assessed through a context-sensitive reconstruction of clinical decision-making that distinguishes adverse outcome, deviation from professional standards, causation, and attribution of liability?

To answer this question, the study combines a structured critical narrative review of the biomedical and medico-legal literature, doctrinal analysis of relevant legal and normative sources, development of CSLAF-OG through a hybrid deductive-inductive process, and exploratory application of the framework to Romanian judicial cases representing different stages of obstetric care. The principal contribution is not the proposition that obstetric liability is multifactorial. Rather, it is the organisation of established clinical, ethical, professional, evidentiary, and organisational factors into an explicit sequential analytical pathway.

## 2. Materials and Methods

### 2.1. Study Design

This study was designed as a structured critical narrative review combined with conceptual framework development, doctrinal legal research, and exploratory case-based application. It was not intended to estimate the prevalence of malpractice, quantify litigation risk, or provide a systematic meta-analysis of medico-legal outcomes. Its purpose was to identify, integrate, and operationalise the principal clinical, ethical, professional, organisational, and legal domains relevant to retrospective assessment of medical liability in obstetric and gynaecological care.

The methodological pathway proceeded from literature search to conceptual evidence synthesis, domain extraction, CSLAF-OG development, exploratory judicial case application, and cross-case interpretation. Four complementary components were combined: a structured search of biomedical and medico-legal literature, targeted examination of legal and professional sources, construction of a context-sensitive liability assessment framework, and exploratory application of that framework to selected Romanian judicial cases.

### 2.2. Research Question and Objectives

The primary research question was: How can medical liability in obstetric and gynaecological care be assessed through a context-sensitive reconstruction of clinical decision-making that distinguishes adverse outcome, deviation from professional standards, causation, and attribution of liability?

Secondary objectives were to identify the clinical, ethical, professional, and organisational domains relevant to such assessment, operationalise these domains within a structured analytical framework, explore the framework across clinically distinct Romanian obstetric cases, and consider its implications for patient safety and healthcare governance.

### 2.3. Literature Search Strategy

A structured literature search was conducted in PubMed/MEDLINE, Scopus, and Web of Science Core Collection, with the final database exports generated on 25 August 2026. The principal search covered publications from 1 January 2015 through the final search date; earlier seminal publications were retained selectively where necessary to establish foundational medico-legal concepts, including malpractice exposure by specialty and defensive medicine [22,23].

The search architecture combined an obstetrics/gynaecology concept block with a malpractice/liability block. Four overlapping thematic searches concerned (2A) clinical judgement and professional standards; (2B) autonomy, informed consent, communication and documentation; (2C) organisational factors, causation and retrospective assessment; and (2D) patient safety, defensive medicine and health-system consequences. The verbatim historical search histories and interface settings were not retained. The strings in Supplementary S3 were reconstructed subsequently from the documented architecture, retained citation exports, date range and recorded yields; they are post hoc approximations and do not establish the exact historical queries or filters.

The broad core searches yielded 452 PubMed, 827 Scopus and 506 Web of Science records (1785 database records before deduplication). The 2A thematic search yielded 69, 187 and 91 records, respectively (347 before deduplication); the yields of 2B–2D are reported separately in Supplementary Table S2. The team combined the overlapping results of 2A–2D. Its legal specialist manually screened the literature using her disciplinary knowledge, and duplicate records were removed, yielding 223 unique publications in the liability-focused thematic corpus. Because the four searches overlap, their yields must not be summed as independent populations. The original exports are retained by the team; the Supplementary Materials (Tables S1 and S2) does not tabulate record-level decisions or separate intermediate exclusion and duplicate counts.

The legal specialist manually assessed the thematic records using her subject expertise and selected publications for conceptual relevance. Publications in languages she could not read were not selected, which may have excluded relevant non-English evidence. The available Supplementary Materials (Tables S1 and S2) does not enumerate exclusions attributable to language, title/abstract or full-text decisions, or duplicate removal. Screening was conducted by this team member rather than independent paired reviewers; no inter-reviewer agreement is claimed. Earlier foundational works were added selectively outside the principal search interval [22,23]. This was a structured critical narrative review, not an exhaustive systematic review.

### 2.4. Evidence Prioritisation and Conceptual Extraction

The legal specialist selected 40 representative anchor publications from the manually curated 223-publication corpus on the basis of her disciplinary judgement. Supplementary Table S1 illustrates the range of obstetric and gynaecological liability topics considered, including claims, clinical standards, communication and documentation, and organisational conditions. The selection was not a formal quality ranking or a prespecified numerical score; its source-by-source decision rules are not tabulated in the Supplementary Tables S1. Reliance on one specialist’s judgement and the language restriction may have influenced the themes represented.

A legal specialist within the research team extracted the information reported in Supplementary Table S1 for the 40 selected anchor publications, including year, database provenance and relevance to eight broad, overlapping conceptual themes. The table records the team’s evidence mapping at that thematic level; it is not a publication-by-thirteen-domain coding matrix or a complete list of the 223 publications.

### 2.5. Development of CSLAF-OG

CSLAF-OG was organised through a hybrid deductive–inductive conceptual synthesis informed by the team’s manual review and the legal specialist’s extraction in Supplementary Table S1. Established medico-legal constructs—contemporaneous information, professional standard, informed consent, documentation, harm, deviation, causation and responsibility—provided deductive starting points. Themes concerning diagnostic uncertainty, reasonable alternatives, capability, communication and organisational conditions informed further interpretation [2,3,4,6,7,8,9,10,11,12,13,14,15,18,19,20,21,24,25,26,27,28,29,30,31,32,33,34,35,36,37,38,39,40,41,42,43,44,45,46,47,48,49,50,51,52,53]. The Supplementary Materials (Tables S1 and S2) records eight broad themes, not an item-level matrix linking every publication to each of the thirteen final domains; the final organisation involved investigator judgement.

The thirteen domains were retained for conceptual distinctiveness and their possible role in retrospective reconstruction. Communication and documentation, capability and system conditions, and deviation and causation were kept separate because conflating each pair would change the analytical question. The resulting sequence places information and alternatives before professional assessment, and attribution after deviation and causation. This is a proposed analytical ordering, not an empirically derived weighting scheme.

CSLAF-OG is conceptual rather than predictive. It does not generate a numerical liability score, assign probabilities of negligence, or constitute a validated medico-legal instrument.

### 2.6. Legal and Normative Source Analysis

The doctrinal component distinguished Romanian statutes and judicial documents from professional guidance, international policy and foreign sources. Romanian statutory provisions were considered in relation to their jurisdiction and temporal scope; later amendments are described as current context and are not used as retrospective standards for earlier clinical events. Official publication and commencement dates were checked for the later enactments cited here [54,55]. Where the cited accounts did not establish an exact clinical event date and the corresponding historical version of a provision, we did not infer the governing text from a later consolidated version.

WHO materials were considered international public-health and quality-of-care guidance [56,57]; ACOG guidance was treated as United States professional guidance rather than Romanian law [58]. Comparative medico-legal sources were used to illuminate concepts, not to determine Romanian liability. Romanian legislation was consulted in official consolidated sources, while publicly accessible anonymised ReJust documents supplied the judicial examples [54,55,59,60,61,62,63,64]. The propositions reported from those documents are distinguished from the authors’ subsequent CSLAF-OG interpretations.

### 2.7. Romanian Case Selection and Framework Application

Romanian judicial documents were identified through the publicly accessible ReJust platform and purposively chosen to illustrate variation across the obstetric continuum. The three principal cases concern prenatal/perinatal risk classification and capability, intrapartum emergency action, and postpartum monitoring and continuity of care [61,62,63]. A fourth case concerning mode of delivery provides an additional illustration [64]. The team has retained the original ReJust search materials and case documents; search strings, access dates and the number of decisions considered are not reported in this manuscript. The team’s legal specialist confirms that all four cited decisions are final. This illustrative selection is not a representative sample of Romanian decisions.

The available anonymised judicial documents were read for reported clinical facts, allegations, expert findings, the court’s recorded reasoning and procedural outcome. Allegations were distinguished from findings in the available document and from the authors’ framework-based interpretation. The available record does not document independent assessors or an adjudication procedure for the domain descriptions in Table 2; these are therefore presented as qualitative author interpretations rather than reproducible ratings.

A qualitative cross-case comparison examined whether the framework’s questions could organise distinct obstetric scenarios. This developer-led, purposive application illustrates possible conceptual use; it is not an independent assessment of face validity, reliability, comparative performance or legal validity.

### 2.8. Ethical Considerations

This study did not involve recruitment of participants, collection of new patient-level data, or intervention involving human subjects. The case-based component relied on anonymised judicial material publicly accessible through ReJust. Accordingly, no research ethics approval or individual patient consent was required for this review and conceptual analysis.

## 3. Results of the Conceptual Evidence Synthesis

The team’s legal specialist manually screened the combined thematic results, and duplicate records were removed to yield 223 unique publications. She selected 40 representative anchor sources and extracted the data mapped in Supplementary Table S1 to eight overlapping broad themes. Within these 40, 38 were assigned to liability/claims, 19 to clinical judgement/standards, 18 to organisation/patient safety, 14 to communication/documentation, eight to diagnosis/monitoring, seven to defensive medicine/intervention, six to consent/autonomy and three to causation/expert assessment. These counts describe the anchor set, not theme prevalence in all 223 publications or a direct publication-level derivation of the thirteen final domains.

### 3.1. Clinical Judgement and Professional Standards

Across the mapped literature, professional standards functioned as important but context-dependent benchmarks. Studies of obstetric practice, forensic review, CTG interpretation, triage, and malpractice claims repeatedly linked liability assessment to applicable guidance, professional competence, and the reasonableness of clinical choices [7,9,13,26,27,29,40,42,49,52]. The evidence did not support treating guidelines as automatic legal rules; their relevance depended on temporal applicability, clinical circumstances, patient-specific risk, and the justification for departure.

This distinction was especially visible in time-sensitive obstetric scenarios. International CTG guidance is not completely uniform [26], while medico-legal analyses of intrapartum monitoring emphasise that a tracing must be interpreted within the clinical context rather than against a single retrospective threshold [42]. Similar considerations apply to triage, operative delivery, and level-of-care decisions [27,29]. The literature therefore supports an analytical question centred on whether the selected course fell outside the range of professionally defensible options at the time, rather than whether another option appears better after the outcome is known.

Qualitative evidence further showed that clinicians integrate standards with experience, risk perception, patient preferences, and institutional constraints [9,13,15]. These findings supported retaining both an ‘applicable professional standard’ domain and a separate ‘clinical judgement and reasonable alternatives’ domain within CSLAF-OG.

### 3.2. Diagnostic Uncertainty, Hindsight Risk, and Reasonable Alternatives

A second recurrent theme was diagnostic and prognostic uncertainty. Malpractice and forensic studies involving ultrasonography, intrapartum imaging, CTG, surgical diagnostic error, and congenital anomaly detection showed that non-detection or delayed recognition can occur under materially different evidentiary conditions [18,19,20,21,28,42]. These studies support separating the question ‘what was ultimately present?’ from ‘what could reasonably have been detected or predicted at the relevant time?’.

Hindsight and outcome bias provide an additional reason for that separation. Experimental and medico-legal literature shows that knowledge of a poor outcome can alter retrospective judgements about earlier clinical decisions [16,17]. In obstetric liability assessment, the risk is particularly pronounced where deterioration is progressive, monitoring is probabilistic, or the causal pathway is multifactorial. Accordingly, CSLAF-OG treats contemporaneous information, uncertainty, and reasonable alternatives as reconstruction domains that must be completed before professional deviation is considered.

The synthesis therefore did not support a binary ‘diagnosis present/diagnosis missed’ model. Instead, it supported a graded reconstruction of detectability, available information, expected professional response, and feasible alternatives, with explicit attention to what was reasonably knowable at the time [18,19,20,21,28,42].

### 3.3. Patient Autonomy and Informed Consent

Patient autonomy emerged as a distinct liability-relevant construct rather than a modifier of technical care. The literature addressed counselling, mode-of-delivery preferences, informed decision-making, refusal, and the tension between patient choice and institutional or professional risk management [15,30,39,41,43,44,48,53,58]. The evidence was most consistent with treating autonomy as a process that can change the set of clinically feasible or ethically acceptable alternatives.

Informed consent was therefore conceptualised as more than documentation of a signature. Relevant evidence included the adequacy and timing of counselling, discussion of material risks and reasonable alternatives, patient understanding, and the management of preference or refusal [41,43,44,53,58]. This justified a dedicated CSLAF-OG domain that is analytically related to communication but not reducible to it.

The literature also cautioned against treating patient preference as either an automatic defence or a subordinate consideration. Context-sensitive assessment must reconstruct what was communicated, what the patient decided, whether that decision was informed, and how it affected the professional options reasonably available at the time.

### 3.4. Communication, Documentation, and Evidentiary Reconstruction

Communication and documentation were among the most recurrent operational themes in the mapped literature [8,10,21,28,32,33,34,35,36,41,43,45,47]. Communication-related allegations appeared across maternity and neonatal care, while quality-improvement studies demonstrated that structured documentation can improve the completeness and retrievability of key delivery information [32,33]. These findings support treating communication failure, implementation failure, and clinical judgement as separable mechanisms.

The distinction matters because an appropriate decision can be implemented late when escalation, handover, referral, or team activation fails [65]. Conversely, communication may be adequate even when the underlying clinical judgement is deficient. Several liability-oriented studies also linked teamwork and interprofessional communication to risk reduction, particularly in high-acuity obstetric settings [34,35,36,45].

Documentation served both clinical and evidentiary functions. Standardised forms for instrumental delivery and shoulder dystocia improved record completeness [32,33], while medico-legal studies repeatedly identified documentation as relevant to later reconstruction [8,10,21,35,41,47]. On this basis, CSLAF-OG treats documentation as an evidentiary mediator: incomplete records do not prove deficient care, but they may limit the ability to establish what was known, communicated, decided, or performed.

### 3.5. Organisational Conditions, Capability, and Patient Safety

The literature also showed that obstetric liability is embedded in organisational systems. Studies described the influence of staffing, teamwork, referral structures, triage, institutional policy, supervision, clinical networks, and quality-improvement programmes on both care processes and liability exposure [8,11,15,27,31,34,36,37,38,39,40,45,51,52]. This evidence supported separating the clinician’s professional capability from the institution’s broader capacity to deliver or escalate care.

System context did not consistently displace individual accountability; its role depends on the circumstances of action and implementation. Complementary evidence outside obstetrics suggests that omissions can be associated with measurable work conditions: in an Italian cross-sectional study of home nursing, missed care was associated, at different levels, with workload, staffing and resource adequacy, leadership, work–life conflict and burnout [66]. These associations neither establish causation nor determine legal responsibility in obstetric practice. Quality-improvement literature also describes using malpractice data to improve systems rather than solely to assign blame [11,34,51].

Accordingly, CSLAF-OG retains two related domains: professional/institutional capability, which asks whether the clinician and facility were competent and equipped for the task, and organisational/system conditions, which asks whether workflow, staffing, escalation, referral, or policy materially affected execution. This distinction enables individual, team, and institutional contributions to be compared without assuming that context necessarily reduces personal responsibility.

### 3.6. Harm, Professional Deviation, Causation, and Attribution

The final recurrent theme was the need to separate harm, professional deviation, causation, and attribution. Forensic and compensation studies showed that severe outcomes and even recognised errors do not map automatically onto findings of liability [6,10,12,24,29,31,40,46,52]. Expert disagreement may occur at the level of professional deviation, causation, or both [6,24,46].

The conceptual implication is sequential: harm identifies the adverse consequence requiring explanation; deviation asks whether conduct fell outside professionally acceptable care; causation asks whether an identified deviation materially contributed to the harm; and attribution asks whose responsibility is supported by the integrated evidence. None of these steps is interchangeable with the others.

This sequence became the liability layer of CSLAF-OG. It also provides an internal safeguard against outcome-driven reasoning because professional deviation is evaluated from reconstructed context and conduct, while the adverse outcome enters separately and converges with deviation only at the causation stage.

## 4. Development and Structure of CSLAF-OG

### 4.1. Framework Derivation from the Evidence Synthesis

The manually curated corpus informed thirteen analytically distinct domains, organised by the authors into four layers. Supplementary Table S1 documents the legal specialist’s extraction and thematic categorisation of 40 selected publications from the 223-publication corpus into eight broad themes. It does not constitute item-level coding of all thirteen final domains. Table 2 gives representative citations and analytical questions; the transition from themes and normative constructs to the final framework remains interpretive.

Four layers were used. Layer I reconstructs the clinical decision context; Layer II identifies professional and autonomy-related expectations; Layer III examines execution and the care environment; and Layer IV addresses outcome, deviation, causation, and attribution. The layers are conceptually ordered but not numerically weighted. A domain may be central in one case and peripheral in another.

**Figure 1:**
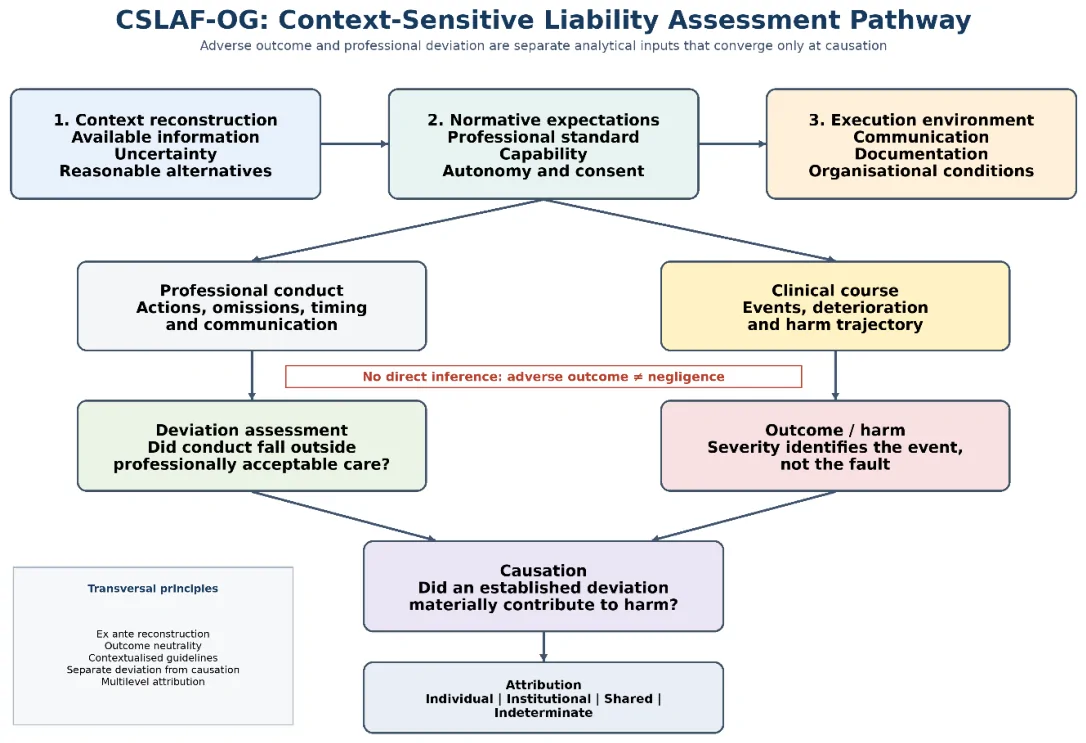
CSLAF-OG analytical pathway. Context reconstruction, normative expectations, and execution environment are analysed before liability attribution. Professional deviation and outcome/harm are treated as distinct inputs that converge only at causation, preventing direct inference from adverse outcome to negligence.

**Figure 2:**
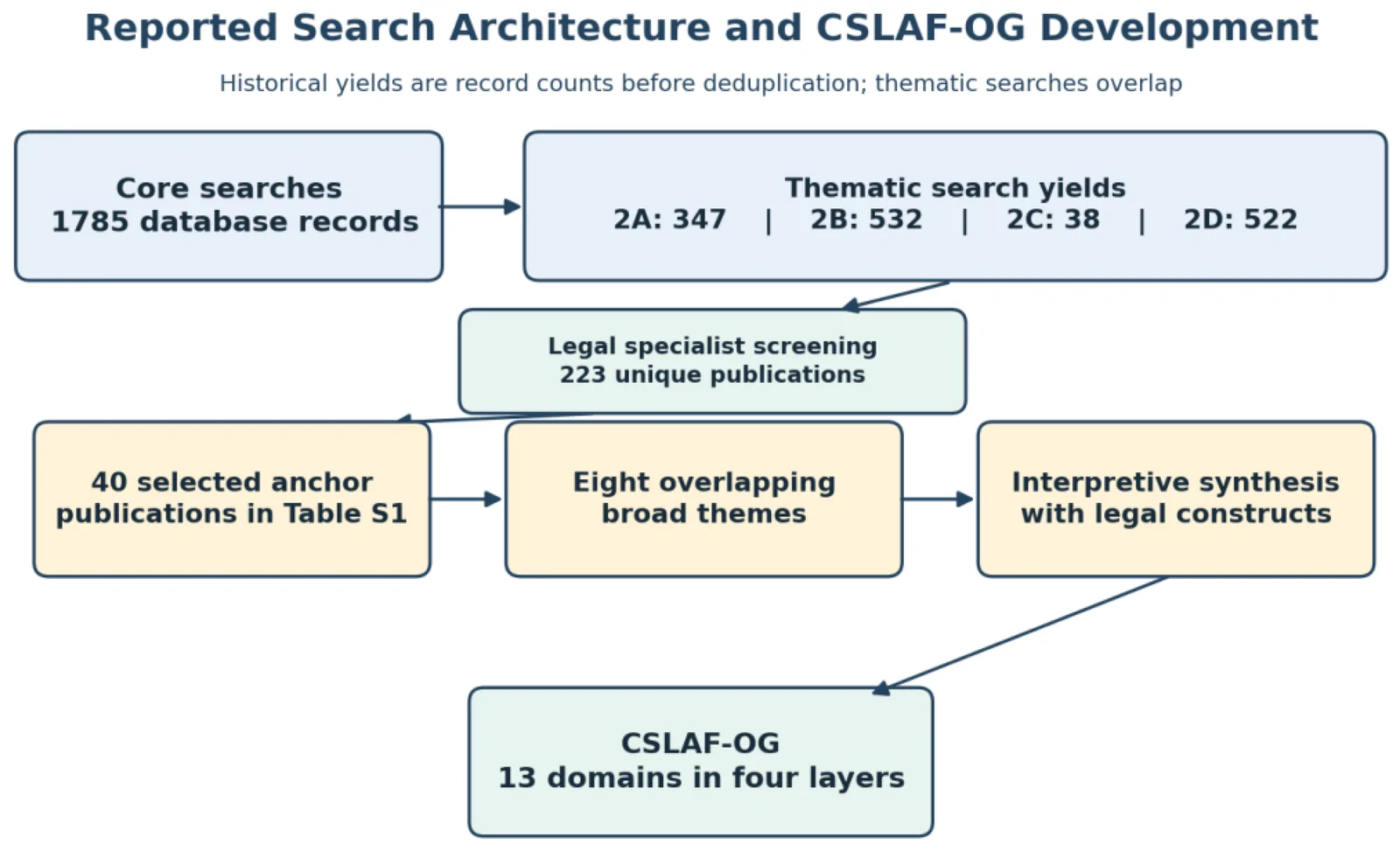
Recorded search architecture and conceptual development of CSLAF-OG. The 347 records belong to search 2A. The team’s legal specialist manually screened the combined, overlapping 2A–2D results using her disciplinary knowledge; after duplicate removal, 223 unique publications were retained. The team retained the exports, although intermediate exclusion counts are not tabulated in the supplement. She extracted the data for 40 selected anchor publications mapped to eight broad themes in Supplementary Table S1; organisation into thirteen final domains was interpretive.

The central design rule is outcome neutrality: an adverse outcome may trigger scrutiny but cannot itself establish professional deviation. Conduct and harm are considered separately before a causation question is asked. Figure 1 shows this proposed analytical logic; Figure 2 distinguishes recorded search yields, the selected anchor table and the subsequent conceptual organisation of CSLAF-OG.

### 4.2. Analytical Domains

Table 2 summarises the thirteen proposed analytical domains, operational questions and illustrative supporting citations. Supplementary Table S1 contains the legal specialist’s extraction for 40 selected publications from the manually curated 223-publication corpus, mapped to eight overlapping broad themes rather than directly to the thirteen final domains.

### 4.3. Analytical Sequence and Transversal Rules

Although the domains are interrelated, CSLAF-OG is intended to be applied sequentially. Analysis begins with reconstruction of the information reasonably available at the time of care and the degree of clinical uncertainty. It then identifies reasonable clinical alternatives and professional expectations applicable to the patient and setting. Patient preferences, communication, documentation, capability, and organisational constraints are subsequently assessed because these factors may alter both the range of feasible options and interpretation of professional conduct.

Only after this reconstruction should the adverse outcome be examined in relation to professional deviation. Where a deviation is identified, causation must be assessed independently. Responsibility is considered at the final stage, after individual and institutional contributions have been distinguished. The sequence can be summarised as: Context → Conduct → Deviation → Causation → Attribution.

CSLAF-OG applies five transversal principles: professional conduct is reconstructed ex ante rather than through hindsight, severity of outcome is not used as a proxy for professional fault, clinical guidelines are treated as contextual benchmarks rather than automatic determinants of liability, professional deviation and causation are assessed as separate questions, and responsibility may arise at individual, team, institutional, or shared levels.

Figure 1 presents the proposed analytical sequence, Table 2 defines its thirteen domains, and Supplementary Table S1 describes thematic assignments for 40 selected publications. These sources serve different purposes and do not establish independent validation of CSLAF-OG.

### 4.4. Interpretation and Limits

Professional standards are deliberately positioned after reconstruction of contemporaneous clinical information and reasonable alternatives because the relevance of a guideline cannot be assessed in isolation from the clinical situation to which it is applied. Diagnostic and prognostic uncertainty is retained as a separate domain because obstetric liability frequently involves retrospective claims that a condition should have been recognised earlier.

CSLAF-OG also separates professional capability from organisational conditions because the same adverse event may involve different levels of responsibility. A clinician may make an appropriate decision that is delayed by institutional constraints, whereas an adequately resourced institution may still experience harm because of deficient individual judgement. In other cases, both levels contribute.

Application of CSLAF-OG may produce non-numerical analytical conclusions such as no relevant professional deviation identified, potential deviation requiring further evidence, professional deviation without sufficiently established causal contribution, professional deviation with evidence supporting causal contribution, or unresolved assessment because of insufficient or conflicting evidence. These are descriptive outputs rather than universal legal categories.

CSLAF-OG is not a validated diagnostic, predictive, or judicial instrument. It does not assign a probability of negligence, determine compensation, replace medico-legal expert evaluation, or supersede national legal standards. Not all thirteen domains carry equal weight in every case.

## 5. Romanian Legal Context for Medical Liability

### 5.1. Patient Rights, Information, and Consent

Romanian law is used in this study to contextualise, not universalise, the framework. Law No. 46/2003 on Patient Rights regulates information, consent and refusal, confidentiality, and continuity of care [59]. For CSLAF-OG, the most relevant elements are the duty to inform the patient about proposed interventions, risks, alternatives, and consequences of non-treatment; the patient’s ability to refuse or discontinue intervention; and the distinct rules applicable in urgent circumstances.

Law No. 27/2026, published on 11 March 2026, subsequently added rights concerning direction and scheduling for further diagnostic evaluation and, under specified conditions, a second medical opinion [54]. These provisions describe later statutory context; they are not applied to clinical events preceding their entry into force.

### 5.2. Professional Liability and Malpractice

Title XVI of Law No. 95/2006 provides the principal statutory framework for civil liability of healthcare professionals and providers [60]. The judicial material examined in this study applies provisions concerning professional error, negligence or imprudence, competence, informed-consent duties, proportional responsibility, and circumstances in which injury may instead be related to accepted complications, resource limitations, or other non-fault factors. CSLAF-OG organises the clinical evidence relevant to these questions but does not replace the statutory legal test.

Romanian courts may use nationally approved specialty guidance or otherwise recognised professional standards when contextualising expected conduct [64]. For framework purposes, such standards are evidentiary benchmarks whose temporal applicability and clinical fit must be established before they are used to assess deviation.

### 5.3. Institutional Responsibility and Professional Duties

Institutional responsibility is also legally relevant. Judicial application of Article 655 (2) of Law No. 95/2006 shows that healthcare providers may bear civil responsibility in relation to harm caused by medical personnel, subject to the version of the law and contractual relationships applicable when the events occurred [60,62,63]. This supports the framework’s separation of individual conduct from institutional contribution.

Professional duties must be distinguished from statutory malpractice rules. The current Code of Medical Deontology of the Romanian College of Physicians entered into force on 1 January 2026 and was subsequently amended [55]. Deontological standards can inform expectations concerning competence, cooperation, communication, and the physician-patient relationship, but their relevance to a historical case depends on temporal applicability and the legal forum in which they are invoked.

### 5.4. Hierarchy of Sources Used in This Study

For the purposes of this study, Romanian statutory law provides the primary domestic legal framework. Romanian judicial decisions are analysed as jurisdiction-specific applications of law to individual disputes. The Romanian Code of Medical Deontology is treated as a binding professional standard within its temporal and institutional scope. WHO documents are used as international ethical and public-health guidance, ACOG recommendations as professional guidance originating in the United States, and foreign medico-legal authorities only as comparative or illustrative material. This hierarchy avoids treating international guidance, foreign case law, professional recommendations, and Romanian law as legally equivalent.

## 6. Application to Romanian Obstetric Jurisprudence

### 6.1. Prenatal/Perinatal Risk Classification, Gestational Dating, and Level-of-Care Capability

Case identification and clinical facts. Decision No. 801/2024 of 13 September 2024, Pitești Court of Appeal, concerned obstetric and neonatal management surrounding caesarean delivery followed by neonatal deterioration and death [61]. The pregnancy records contained material discrepancies concerning gestational age. The obstetrician documented 39 weeks at delivery, while other evidence considered by the court supported a lower gestational age and raised the possibility of prematurity. The maternity unit was level 1b and did not have neonatal intensive-care beds; evidence before the court indicated that delivery below 37 completed weeks required care at a higher-level unit.

Alleged professional failure and expert evidence. The disputed conduct therefore extended beyond gestational dating to risk classification, choice of delivery setting, communication of gestational information, neonatal capability, and timing of transfer. Medico-legal opinions were not fully concordant on causation. Some INML experts did not express certainty in the absence of a forensic autopsy, whereas another forensic expert considered the identified obstetric/neonatal conduct causally related to the death [61].

Legal issue, court reasoning, and outcome. The appellate court evaluated both the obstetric decision pathway and the neonatal response. It ultimately treated the conduct of both defendants as causally relevant to the neonatal death and resolved the civil-liability component accordingly [61]. The case is therefore not reducible to a single disputed clinical act; it involved longitudinal information, institutional capability, referral hierarchy, and expert disagreement over causal contribution.

CSLAF-OG relevance and limitation. The case places primary weight on contemporaneous information, diagnostic/prognostic uncertainty, professional standard, capability, documentation, system conditions, causation, and multilevel attribution. Its principal limitation for framework testing is the complexity of the causal record, including disagreement among experts and the absence of a medico-legal autopsy.

### 6.2. Intrapartum Emergency Decision-Making and Delayed Caesarean Delivery

Case identification and clinical facts. Decision No. 970/2026 of 28 May 2026, București Court of Appeal, concerned acute fetal compromise during labour [62]. The record documented fetal heart rates of 136 beats/min at 12:00, 125 at 12:30, 180 at 13:00, and 138 at 13:25, followed at 13:30 by late decelerations to approximately 90 beats/min. Oxytocin was stopped, maternal repositioning was initiated, and caesarean delivery was indicated. Extraction occurred at 14:18.

Alleged professional failure and expert evidence. The central allegation was not the technical performance of caesarean delivery but delay after recognition of the emergency. The record placed anaesthesia consultation at approximately 13:50, theatre arrival at 14:00, spinal anaesthesia by about 14:05, operative start near 14:10, and extraction at 14:18. Expert evidence linked the prolonged interval to fetal hypoxia and severe permanent neurological injury [62].

Legal issue, court reasoning, and outcome. The court treated the guideline recommendation to perform emergency caesarean delivery as rapidly as possible, with a sub-30-min interval described as a desired benchmark, rather than as a mechanical legal threshold. It also examined organisational contribution and found that anaesthesia and neonatology responded promptly once activated, supporting attribution predominantly to delayed clinician action while also recognising a basis for institutional civil responsibility. Criminal proceedings had ended because limitation intervened; the civil component was adjudicated on appeal [62].

CSLAF-OG relevance and limitation. The illustration separates recognition of fetal compromise at 13:30 from the timing of escalation, preparation and extraction at 14:18. The sub-30-min recommendation is considered a desired benchmark within this chronology, not a self-executing legal threshold. The reported response of anaesthesia and neonatology once activated helps locate the alleged delay in the sequence without making the adverse neurological outcome or elapsed time alone proof of deviation. Expert evidence concerning causation and the court’s recorded allocation of civil responsibility remain distinct questions. This illustrates the framework’s ordering; it does not establish superiority over conventional case analysis.

### 6.3. Postpartum Monitoring, Discharge, and Continuity of Care

Case identification and clinical facts. Decision No. 779/2025 of 26 June 2025, Ploiești Court of Appeal, concerned a patient who developed persistent abdominal pain and fever after caesarean delivery, was discharged with ongoing symptoms, subsequently deteriorated, and required emergency surgery for peritonitis and septic shock, including hysterectomy and removal of the right adnexa [63].

Alleged professional failure and expert evidence. The operative technique itself was analytically distinguished from the broader perioperative duty. Expert evidence focused on inadequate postoperative monitoring, underestimation of fever and abdominal symptoms, discharge despite a pathological clinical course, lack of appropriate multidisciplinary reassessment, and the need to reconsider antimicrobial treatment [63].

Legal issue, reasoning reported in the cited document, and outcome. The final judgment cited from ReJust describes a continuity-of-care analysis under Law No. 46/2003 and reports a causal link between deficient postoperative care and the subsequent life-saving surgery with irreversible loss of reproductive capacity [63].

CSLAF-OG interpretation and limitation. The cited account makes postoperative information, reassessment, documentation, continuity, system conditions, harm, possible deviation and causation relevant to the framework. The proposed allocation of individual and institutional contributions is an author interpretation of that account, not an additional judicial finding.

### 6.4. Secondary Illustrative Case: Mode of Delivery and Accepted Complications

Case identification and clinical facts. Decision No. 279/2025 of 20 November 2025, Cluj Court of Appeal, is used as a secondary negative illustration rather than as one of the three principal cross-case cases [64]. The underlying civil action alleged, among other matters, failure to indicate caesarean delivery and defective management of vaginal birth. Medico-legal evidence found no documented caesarean indication, considered vaginal delivery appropriate, and characterised the perineal injury as a recognised possible complication.

Legal reasoning and framework relevance. The first-instance analysis also rejected reliance on a 2020 ministerial order to assess a birth that occurred in 2017, illustrating the importance of temporally appropriate standards. The appellate proceedings were limited principally to legal costs and did not reopen the merits of the malpractice findings [64]. For CSLAF-OG, the case is useful because the same framework can support a conclusion of no relevant professional deviation when adverse outcome, recognised complication, contemporaneous standard, consent, and causation are considered separately.

## 7. Cross-Case Analysis

The comparative application of CSLAF-OG across the three principal Romanian cases is summarised analytically in Table 3.

**Table 3:** Illustrative cross-case interpretation of CSLAF-OG across three Romanian judicial documents. ‘Central’, ‘supporting’ and ‘limited’ describe the authors’ qualitative reading of domain relevance; they are neither scores nor findings by the courts.

CSLAF-OG Domain	Decision 801/2024	Decision 970/2026	Decision 779/2025	Cross-Case Interpretation
Contemporaneous information	Central—Gestational-age data and longitudinal discrepancies	Central—Evolving CTG and documented bradycardia	Central—Fever, pain, postoperative deterioration	Liability analysis begins with what was known at the relevant time.
Diagnostic/prognostic uncertainty	Central—Gestational dating and prematurity	Central—Timing and severity of fetal compromise	Central—Expected recovery versus infection	Uncertainty differs by phase but must precede hindsight assessment.
Clinical judgement	Central—Timing/place of delivery and referral	Central—Recognition plus implementation of urgent delivery	Central—Observation/investigation versus discharge	The disputed decision differs, but the same analytical sequence applies.
Professional standard	Central—Gestational-age and level-of-care standards	Central—Emergency caesarean guidance	Central—Postoperative surveillance and continuity	Standards must be temporally appropriate and case-specific.
Capability	Central—Level 1b unit without NICU beds	Supporting—Theatre, anaesthesia, neonatology investigated	Supporting—Access to multidisciplinary diagnostic care	Capability modifies interpretation and attribution.
Autonomy/consent	Limited—Secondary	Limited—Secondary once emergency established	Limited—Relevant to emergency surgery context	Not every domain carries equal relevance in every case.
Communication	Supporting—Gestational-risk information and handover	Central—Repeated midwife escalation and emergency-team activation	Supporting—Symptoms communicated to staff and continuity issues	Communication may affect implementation independently of judgement.
Documentation	Central—Gestational age central	Central—CTG and timestamps central	Central—Fever and discharge documentation central	Documentation mediates retrospective reconstruction in all cases.
System conditions	Central—Referral hierarchy central	Supporting—System response largely prompt once activated	Central—Continuity and discharge process relevant	Contextual analysis does not automatically dilute individual accountability.
Harm	Central—Neonatal death	Central—Permanent severe neurological injury	Central—Sepsis, hysterectomy, irreversible infertility	Severity identifies harm, not fault.
Deviation	Central—Court-supported conduct concerns	Central—Delay supported by judicial and expert analysis	Central—Postoperative omission supported by expert evidence	Deviation must be established independently of harm.
Causation	Central—Expert disagreement existed before appellate resolution	Central—expert-supported causal finding recorded in the judicial document	Central—a causal finding is reported in the cited account	Causation is a separate analytical stage.
Attribution	Central—Multiple professionals and care pathway	Central—Predominantly individual conduct plus institutional civil responsibility	Central—author interpretation of individual and hospital roles in the cited account	Attribution follows integrated analysis of individual and system contributions.

Note: The descriptions of domain relevance have no validated thresholds, numerical weight or claim of inter-rater agreement. The table distinguishes the content of the cited judicial accounts from the authors’ interpretation.

The three illustrations emphasise different questions. Decision No. 801/2024 raises longitudinal information, gestational-risk interpretation, capability and referral; Decision No. 970/2026 highlights timing, monitoring, escalation and implementation; Table 3 describes author-assigned domain relevance, without numerical or validated weights.

Across the examples, the proposed sequence asks for contemporaneous context and conduct before considering harm, deviation, causation and attribution. Contextual analysis can point towards individual, organisational or mixed contributions, but these purposively selected cases do not establish how often each configuration occurs or whether the framework improves decisions.

The secondary Cluj case illustrates that an adverse outcome may coexist with an available judicial account that did not establish a relevant professional deviation. Taken together, these developer-selected examples show how CSLAF-OG can organise different narratives; they do not provide independent face-validity, reliability or performance evidence.

## 8. Discussion

### 8.1. Principal Interpretation of the Framework

This study extends the malpractice literature by converting recurrent liability-related themes into an ordered analytical process. The framework’s main contribution is not a new definition of negligence but a disciplined sequence: reconstruct what was knowable and feasible, identify the professional and autonomy-related expectations, examine how care was executed, and only then analyse deviation, harm, causation, and responsibility. This sequence is intended to reduce the risk that outcome severity becomes an implicit shortcut for fault.

In the intrapartum illustration, separating recognition of fetal compromise from the subsequent activation and response of the team helps locate the alleged delay without treating a 30-min benchmark as a legal cut-off. The prenatal/perinatal and postpartum examples raise different questions about capability, referral or continuity. These observations describe the available case narratives and author interpretation, not a tested advantage over other approaches.

### 8.2. Relationship to Existing Literature and Incremental Contribution

The 2025 JCM review by Toma-Tumbar et al. established that obstetric and gynaecological malpractice is shaped by standards of care, diagnostic errors, complications, counselling, informed consent, surgical issues, documentation, and multiple stakeholders [1]. The present findings are consistent with that landscape. The incremental contribution is narrower but more operational: CSLAF-OG specifies the order in which these domains should be interrogated and makes three distinctions explicit—ex ante reconstruction versus hindsight, deviation versus outcome, and causation versus final attribution (Table 1).

The broader database-derived literature supports those distinctions. Studies of clinical judgement and risk perception show that decision-making is negotiated within professional and organisational constraints [9,13,15]; documentation and communication studies show that later reconstruction depends on what was recorded and transmitted [8,32,33,34,35,36,41,43,45]; and medico-legal series show that severe harm, professional error, expert opinion, and legal responsibility do not necessarily coincide [6,10,12,24,29,31,40,46,52]. The framework integrates these findings rather than treating them as independent risk-factor lists.

### 8.3. Patient Safety and Organisational Implications

Medical liability systems influence healthcare beyond adjudication of individual claims. Litigation and perceived liability risk can affect clinical behaviour, documentation, willingness to deviate from guidelines, escalation practices, workforce confidence, resource allocation, and organisational learning [8,11,14,34]. These effects are especially relevant in maternity care, where severe outcomes and prolonged legal processes can reinforce defensive or blame-oriented cultures.

Defensive medicine represents one such pathway. High-risk specialists report additional testing, intervention, and avoidance behaviour in response to liability concerns [14,23]. Obstetric-specific literature also links medico-legal pressure and perceived risk to mode-of-delivery decision-making [9,38,39,67]. These studies demonstrate association and professional perception; they do not establish that liability pressure independently causes a particular caesarean rate or intervention.

For patient safety, the value of contextual reconstruction is that it can identify preventable process weaknesses even when individual negligence is not established. Communication failure, inadequate escalation, incomplete documentation, insufficient capability, and referral barriers can remain legitimate safety targets regardless of the final legal outcome [11,34,36,45,51].

Legal accountability and safety improvement have overlapping but distinct purposes. An organisation may identify processes requiring improvement even where individual liability has not been established. CSLAF-OG proposes to consider those questions separately and to reserve legal attribution for the applicable jurisdictional assessment.

Respectful maternity care remains relevant to this broader safety context because deficiencies in communication, autonomy, consent, dignity, or institutional culture may independently harm patients and erode trust [68,69,70]. The legal recognition of concepts such as obstetric violence varies across jurisdictions; CSLAF-OG therefore does not treat the label itself as a category of malpractice but analyses the underlying conduct through the relevant autonomy, communication, documentation, and organisational domains.

### 8.4. Implications for Clinical Practice, Policy, and Research

For clinical practice, CSLAF-OG supports contemporaneous documentation of the information available at key decision points, reasonable alternatives considered, rationale for departures from standard recommendations, patient preferences, and interprofessional communication. The purpose is continuity and reconstructability of care, not defensive documentation for its own sake [8,32,33,43].

For healthcare organisations, liability prevention should extend beyond individual compliance. Institutions should evaluate whether clinicians have the competence, personnel, equipment, escalation pathways, and referral capacity necessary to meet expected standards and whether adverse-event review distinguishes decision failure from implementation or system failure [11,27,34,36,45,51].

For medico-legal evaluation, CSLAF-OG may serve as an evidence-organising scaffold while leaving substantive legal standards to the relevant jurisdiction. It should not be presented as a legal test, scoring instrument, or substitute for expert opinion.

Future research should test the framework prospectively or retrospectively in larger, independently assessed case series. Priority questions include inter-rater agreement, completeness of domain capture, whether structured ex ante reconstruction reduces hindsight-related disagreement, and whether the framework improves discrimination between individual and organisational contributions. External testing should include both liability-positive and liability-negative cases across different legal systems.

## 9. Limitations

Several limitations bear directly on interpretation. This structured critical narrative review was not designed for exhaustive study inclusion or effect synthesis. One legal specialist manually screened the overlapping thematic search outputs using her disciplinary judgement; she did not select publications in languages she could not read. The resulting language restriction and single-selector judgement may have shaped the corpus and the 40 anchor publications. The team retained the exports, but the supplementary record does not tabulate intermediate exclusion and duplicate counts or record-level selection decisions. The verbatim historical bibliographic query strings and interface settings were not retained, so reconstructed strings in Supplementary S3 cannot replicate those searches verbatim. The specialist’s extraction maps 40 selected publications to eight broad themes, not each publication to all thirteen final domains.

CSLAF-OG has not undergone independent face-validity assessment, empirical reliability testing or comparative evaluation. Developer-led application to purposively chosen judicial cases illustrates possible use only; it does not establish analytical usability as a measured property, predictive validity or legal validity.

The Romanian judicial examples were purposively selected across prenatal/perinatal, intrapartum and postpartum care. The team retained its ReJust search materials and case documents, but the search terms, screening denominator and case-selection log are not yet reported here; the authors’ illustrative domain descriptions were not independently rated. The examples cannot represent Romanian malpractice jurisprudence or support prevalence or performance claims.

Fourth, legal concepts, evidentiary thresholds, causation standards, and the authority of guidelines vary across jurisdictions. Romanian statutes and case law were therefore distinguished from international professional guidance and foreign medico-legal literature throughout the analysis.

The case analyses are confined to the clinical and expert material reported in the cited judicial documents. Official sources confirm that Law No. 27/2026 was published on 11 March 2026 and that the current Code of Medical Deontology entered into force on 1 January 2026 [54,55]; they are presented as later context, not retrospective standards for earlier clinical events.

Finally, the framework does not quantify the relative contribution of its domains, prescribe a burden of proof, or resolve competing expert evidence. Its utility will depend on whether future empirical work shows that structured reconstruction improves consistency, transparency, or explanatory value in real medico-legal assessment.

## 10. Conclusions

Medical liability in obstetric and gynaecological care should not be reconstructed backwards from the severity of an adverse outcome. The evidence reviewed here supports beginning with contemporaneous information, uncertainty, realistic alternatives, professional expectations, autonomy, communication, documentation, capability, and organisational conditions.

CSLAF-OG organises these elements into thirteen domains across four layers and then separates professional deviation from outcome, causation, and final attribution. Its novelty lies in this ordering and integration, not in claiming that the underlying liability-related factors are individually new.

Illustrative application to prenatal/perinatal, intrapartum and postpartum judicial narratives shows how different domains may become relevant whilst the same questions are posed in sequence. The examples suggest how context may inform discussion of individual and institutional contributions, but cannot establish an improvement in attribution or agreement.

CSLAF-OG should be regarded as a conceptual analytical scaffold. Independent assessors, broader and mixed-outcome case sets, and documented coding procedures are needed to examine its completeness, inter-rater agreement and possible contribution to reducing hindsight-driven disagreement.

## Figures and Tables

**Table 1:** Relationship between the 2025 JCM narrative review and the present study.

Analytical Feature	Toma-Tumbar et al. 2025 [1]	Present Study
Primary purpose	Narrative overview of malpractice drivers, stakeholders, expectations, and legal/clinical implications	Operationalise retrospective liability assessment through a context-sensitive analytical sequence
Evidence base	Narrative review of 25 articles	Three-database structured critical narrative review plus doctrinal legal analysis and judicial case application
Core organising logic	Topical discussion of standards, diagnostic errors, counselling, procedures, consent, documentation, and litigation	Four analytical layers and thirteen domains progressing from context and conduct to deviation, causation, and attribution
Temporal perspective	No explicit ex ante reconstruction framework	Explicit reconstruction of information, uncertainty, alternatives, and capability available at the time of care
Outcome, deviation, and causation	Discussed within the broader malpractice landscape	Explicitly separated as distinct analytical stages; outcome is not used as a proxy for deviation
Individual versus organisational responsibility	Recognises multiple stakeholders and system implications	Multilevel attribution is an explicit final step: individual, institutional, shared, or indeterminate
Case-law application	No structured cross-case framework application	Exploratory application to three principal Romanian obstetric decisions plus one secondary illustrative case

**Table 2:** CSLAF-OG analytical domains, primary questions, evidentiary focus, and interpretive safeguards.

Layer/Domain	Primary Analytical Question	Illustrative Evidence	Interpretive Caution
Layer I: Contemporaneous clinical information	What information was reasonably available at the relevant time?	Clinical records, monitoring, investigations, chronology [6,20,27,28,45]	Later knowledge should not be retroactively attributed
Layer I: Diagnostic and prognostic uncertainty	What could reasonably have been detected, predicted, or excluded?	Diagnostic performance, clinical limitations, patient-specific factors [18,19,20,21,28,42]	Missed diagnosis or unexpected deterioration does not automatically establish deviation
Layer I: Clinical judgement and reasonable alternatives	Which options were professionally defensible at that time?	Clinical reasoning, alternatives, consultations, expert evidence [7,9,13,15,27,29,40,49]	Another retrospectively preferable option does not itself establish negligence
Layer II: Applicable professional standard	Which guideline, protocol, or professional expectation applied?	Contemporaneous guidelines, national protocols, institutional standards [7,25,26,29,42,49,52]	Guidelines are evidentiary benchmarks, not automatic legal rules
Layer II: Professional and institutional capability	Were clinician and institution appropriately competent and equipped?	Training, credentialing, staffing, equipment, referral capacity [10,27,36,45,51,52]	Resource limitations neither automatically excuse nor automatically establish liability
Layer II: Patient autonomy and informed consent	What information, preferences, refusal, or consent issues affected the decision?	Counselling, consent documentation, patient preferences [15,30,39,41,43,44,48,53,58]	A signed form is not equivalent to a complete consent process
Layer III: Communication	Was clinically relevant information communicated appropriately and in time?	Handover, escalation, counselling, team communication [8,28,35,36,41,43,45]	Communication failure should be distinguished from underlying clinical judgement
Layer III: Documentation	Can the clinical reasoning and actions be reconstructed reliably?	Medical notes, timestamps, reports, imaging records [8,21,32,33,34,35,41]	Poor documentation does not necessarily prove poor care, but may create evidentiary uncertainty
Layer III: Organisational and system conditions	Did system-level constraints materially affect care?	Staffing, workload, theatre access, referral pathways, institutional policy [8,11,15,31,34,36,37,38,45,51]	Institutional contribution should be assessed separately from individual conduct
Layer IV: Outcome and harm	What adverse clinical consequence occurred?	Clinical outcome, severity, duration, sequelae [6,10,12,31,36,46,47]	Severity of harm is not a proxy for severity of fault
Layer IV: Deviation analysis	Did conduct fall outside professionally acceptable care?	Standards, context, expert assessment [6,7,24,25,29,40,42,52]	Deviation must be established independently of outcome
Layer IV: Causation	Did the alleged deviation materially contribute to harm?	Timeline, pathophysiology, expert evidence, counterfactual reasoning [6,24,29,46]	Causation remains jurisdiction-dependent and separate from breach
Layer IV: Attribution of responsibility	Is responsibility individual, institutional, shared, uncertain, or unsupported?	Integrated findings from preceding domains [2,3,6,10,12,45,52]	Attribution should be the final analytical stage

## Data Availability

No new datasets were generated. The anonymised judicial decisions analysed in this study are publicly available through the Romanian ReJust database at the sources cited in References [61,62,63,64].

## Supplementary Materials

The following supporting information can be downloaded at: https://www.mdpi.com/article/10.3390/healthcare14203531/s1, Supplementary Table S1: the legal specialist’s extraction and thematic mapping of 40 selected anchor publications from the 223-publication corpus; Supplementary Table S2: historical search yields and reconstructed strategy identifiers; Supplementary S3: post hoc reconstructed database-specific strategies and language-restriction audit.

## Abbreviations

The following abbreviations are used in this manuscript:

ACOG	American College of Obstetricians and Gynecologists
CSLAF-OG	Context-Sensitive Liability Assessment Framework for Obstetrics and Gynaecology
CTG	Cardiotocography
INML	National Institute of Legal Medicine (Romania)
NICU	Neonatal intensive care unit
WHO	World Health Organization
