# Supplementary material for: Beyond Individual Fault: A Context-Sensitive Framework for Assessing Medical Liability in Obstetrics and Gynaecology
Source: Healthcare (Basel). 2026 Oct 20;14(20):3531. doi: 10.3390/healthcare14203531 (PMC13704618; doi:10.3390/healthcare14203531)
Supplement: Supplementary file 1 [file healthcare-14-03531-s001.zip › healthcare-4609910-supplementary.pdf]

Supplementary Materials — CSLAF-OG Thematic Mapping and Search Record

Supplementary Table S1 presents information extracted by the research team's legal specialist for 40 anchor publications selected from the manually screened and deduplicated 223-publication corpus formed from the overlapping thematic searches 2A–2D. The specialist screened using her disciplinary knowledge and did not select publications in languages she could not read. The publications are assigned to eight broad conceptual themes; these overlapping assignments are not an original source-by-thirteen-domain matrix.

Supplementary Table S1. Forty Selected Publications Mapped to Eight Broad Conceptual Themes

Ref.	Year	Publication	Database(s)	Primary CSLAF-OG relevance
**6**	2024	Forensic Obstetrics and Clinical Risk Factors<br>DOI: 10.1055/a-2412-3855	PubMed, Scopus, WoS	Causation/expert assessment; Liability/claims; Defensive medicine/intervention
**7**	2016	Litigations and the Obstetrician in Clinical Practice.<br>DOI: 10.4103/2141-9248.181847	PubMed, WoS	Liability/claims; Clinical judgement/standards; Communication/documentation
**8**	2016	An exploration of the effects of clinical negligence litigation on the practice of midwives in England: A phenomenological study<br>DOI: 10.1016/j.midw.2015.10.005	PubMed, Scopus, WoS	Liability/claims; Organisation/patient safety; Clinical judgement/standards
**9**	2018	Clinicians' views of factors influencing decision-making for caesarean section: A systematic review and metasynthesis of qualitative, quantitative and mixed methods studies<br>DOI: 10.1371/journal.pone.0200941	PubMed, Scopus, WoS	Defensive medicine/intervention; Clinical judgement/standards; Organisation/patient safety
**10**	2016	Obstetric and gynecologic malpractice claims in Saudi Arabia: Incidence and cause<br>DOI: 10.1016/j.jflm.2016.02.001	Scopus	Liability/claims; Defensive medicine/intervention; Communication/documentation
**11**	2019	Malpractice litigation, quality improvement, and the University Hospitals Obstetric Quality Network<br>DOI: 10.1177/2516043519877330	Scopus, WoS	Liability/claims; Organisation/patient safety; Clinical judgement/standards
**12**	2020	Analysis of professional liability claims with catastrophic payouts in gynecology and obstetrics	Scopus	Liability/claims; Organisation/patient safety
**14**	2021	Physicians? views and experiences of defensive medicine: An international review of empirical research<br>DOI: 10.1016/j.healthpol.2021.02.005	PubMed, Scopus, WoS	Defensive medicine/intervention; Liability/claims; Communication/documentation
**15**	2017	A qualitative exploration of how midwives' and obstetricians' perception of risk affects care practices for low-risk women and normal birth<br>DOI: 10.1016/j.wombi.2017.02.005	Scopus	Organisation/patient safety; Liability/claims; Consent/autonomy
**19**	2019	Intrapartum sonography of fetal head in second stage of labor with neuraxial analgesia: A literature review and	PubMed, Scopus	Liability/claims; Diagnosis/monitoring; Clinical judgement/standards

		possible medicolegal aftermath<br>DOI: 10.26355/eurrev_201904_17673		
**20**	2025	Antenatal ultrasound cannot detect all congenital Anomalies: Clinical limitations, India-specific barriers, and medicolegal perspectives<br>DOI: 10.1016/j.jham.2025.100331	PubMed, Scopus, WoS	Diagnosis/monitoring; Liability/claims; Clinical judgement/standards
**21**	2021	An Ounce of Prevention: A Review of the Most Common Errors in OB/GYN Ultrasound Leading to Litigation and How to Avoid Them<br>DOI: 10.5005/jp-journals-10009-1817	Scopus	Diagnosis/monitoring; Liability/claims; Communication/documentation
**26**	2018	Comparison of International Guidelines for the Application of Cardiotocography<br>DOI: 10.1055/s-0043-122945	Scopus	Diagnosis/monitoring; Clinical judgement/standards; Liability/claims
**27**	2016	Contemporary obstetric triage<br>DOI: 10.1097/ogx.0000000000000282	PubMed, Scopus, WoS	Liability/claims; Clinical judgement/standards; Organisation/patient safety
**28**	2024	Characteristics and contributing factors of diagnostic error in surgery: analysis of closed medico-legal cases and complaints in Canada<br>DOI: 10.1503/cjs.003523	PubMed, Scopus, WoS	Diagnosis/monitoring; Liability/claims; Communication/documentation
**29**	2017	Evidence-based draft guideline for prevention of midwifery malpractices based on referred cases to the forensic medicine commission and the medical council from 2006-2011<br>DOI: 10.4103/ijnmr.ijnmr_75_16	PubMed, Scopus	Liability/claims; Clinical judgement/standards; Causation/expert assessment
**30**	2015	Medicolegal issues in labor analgesia<br>DOI: 10.1007/978-3-319-13890-9_25	Scopus	Liability/claims; Clinical judgement/standards; Consent/autonomy
**31**	2019	Approved claims for compensation from gynecological patients in Norway—What characterizes the cases? A 14-year nationwide study<br>DOI: 10.1111/aogs.13605	PubMed, Scopus	Liability/claims; Clinical judgement/standards; Organisation/patient safety
**32**	2016	Documentation of instrumental vaginal deliveries: The benefits of a pro forma<br>DOI: 10.1177/1833358316647547	PubMed, Scopus, WoS	Communication/documentation; Clinical judgement/standards; Liability/claims
**33**	2020	Effect of Implementing a Standardized Shoulder Dystocia Documentation Form on Quality of Delivery Notes<br>DOI: 10.1097/pts.0000000000000305	Scopus	Communication/documentation; Liability/claims
**34**	2019	Sustaining and spreading quality improvement: Decreasing intrapartum malpractice risk<br>DOI: 10.1002/jhrm.21329	PubMed, Scopus	Liability/claims; Communication/documentation; Organisation/patient safety
**35**	2016	Committee Opinion No. 681: Disclosure and Discussion of Adverse Events<br>DOI: 10.1097/aog.0000000000001816	Scopus	Communication/documentation; Liability/claims; Organisation/patient safety
**36**	2019	The most common patient safety issues resulting in legal action against	Scopus	Liability/claims; Organisation/patient safety; Communication/documentation

		neonatologists<br>DOI: 10.1053/j.semperi.2019.08.010		
**37**	2018	Risk and boundary work in contemporary maternity care: tensions and consequences<br>DOI: 10.1080/13698575.2017.1398820	Scopus	Organisation/patient safety; Liability/claims
**38**	2017	Barriers and enablers in the implementation of a program to reduce cesarean deliveries<br>DOI: 10.1186/s12978-017-0369-3	Scopus, WoS	Defensive medicine/intervention; Liability/claims; Organisation/patient safety
**39**	2017	Do Women Have a Choice? Care Providers' and Decision Makers' Perspectives on Barriers to Access of Health Services for Birth after a Previous Cesarean<br>DOI: 10.1111/birt.12270	Scopus, WoS	Defensive medicine/intervention; Organisation/patient safety; Liability/claims
**40**	2019	Medical malpractice in Iran: A systematic review<br>DOI: 10.34171/mjiri.33.110	PubMed, Scopus	Liability/claims; Clinical judgement/standards; Organisation/patient safety
**41**	2025	Risk factors and management of medical disputes: An analysis of preliminary appraisal reports<br>DOI: 10.1371/journal.pone.0334917	PubMed, Scopus, WoS	Liability/claims; Consent/autonomy; Communication/documentation
**42**	2017	Submitting medico-legal intra-partum CTG (I-P CTG) monitoring to the Bolam and Bolitho principles.<br>DOI: 10.1177/0025817216683639	PubMed, Scopus	Liability/claims; Diagnosis/monitoring
**43**	2026	Perspectives and practices of counselling, consent and debriefing for cesarean sections: a mixed-methods study in two Indian states<br>DOI: 10.1186/s12884-026-09150-w	PubMed, Scopus, WoS	Consent/autonomy; Communication/documentation; Clinical judgement/standards
**44**	2022	Midwives' experiences of facilitating informed decision-making - a narrative literature review<br>DOI: 10.1016/j.midw.2022.103322	PubMed, WoS	Clinical judgement/standards; Liability/claims; Organisation/patient safety
**45**	2017	Obstetric Anesthesia Liability Concerns<br>DOI: 10.1097/grf.0000000000000272	PubMed, Scopus, WoS	Liability/claims; Communication/documentation; Organisation/patient safety
**46**	2018	Pregnancy-associated death - Clarifying the cause of death and medico-legal assessments in accusations of malpractice<br>DOI: 10.1055/s-0043-120446	Scopus	Liability/claims; Causation/expert assessment
**47**	2021	Intraoperative injuries from abdominopelvic surgery: an analysis of national medicolegal data<br>DOI: 10.1503/cjs.010219	WoS	Liability/claims; Communication/documentation; Clinical judgement/standards
**48**	2023	Medical malpractice in robotic surgery: a Westlaw database analysis<br>DOI: 10.1007/s11701-022-01417-6	PubMed, Scopus, WoS	Liability/claims; Consent/autonomy; Diagnosis/monitoring
**49**	2019	Kristeller maneuvers or fundal pressure and maternal/neonatal morbidity:	PubMed, Scopus, WoS	Liability/claims; Clinical judgement/standards

		obstetric and judicial literature review DOI: 10.1080/14767058.2018.1441278		
**50**	2020	Characteristics of Paid Malpractice Claims among Resident Physicians from 2001 to 2015 in the United States DOI: 10.1097/acm.0000000000003039	PubMed, Scopus, WoS	Liability/claims; Clinical judgement/standards
**51**	2026	Detection of obstetric anal sphincter injuries: From the fear of malpractice to a quality indicator of a center DOI: 10.1002/ijgo.70415	PubMed, Scopus, WoS	Organisation/patient safety; Liability/claims; Diagnosis/monitoring
**52**	2026	Criminal liability in medical malpractice cases in Jordan: Analysis DOI: 10.1002/jhrm.70031	PubMed, Scopus	Liability/claims; Organisation/patient safety; Clinical judgement/standards
**53**	2015	Communicating risks in obstetrics: the example of elective cesarean section DOI: 10.1007/s00129-015-3715-y	Scopus, WoS	Consent/autonomy; Defensive medicine/intervention; Liability/claims

Note: The team's legal specialist manually selected 40 representative publications from the 223 unique publications, then extracted the year, database provenance and thematic relevance shown here. Her disciplinary judgement informed the selection. Publications in languages she could not read were not selected; the number excluded for this reason is not tabulated. The eight themes are not identical to the thirteen final CSLAF-OG domains, and their frequencies refer only to the 40-source anchor set.

Supplementary Table S2. Recorded Historical Search Yields and Post Hoc Strategy Identifiers

The database exports dated 25 August 2026 were retained by the team. Core-search yields total 1,785 records before deduplication. The 347 records (69 PubMed, 187 Scopus, 91 Web of Science) belong specifically to thematic search 2A. The team's legal specialist manually screened the combined overlapping records from 2A–2D; after duplicate removal, 223 unique publications were retained. The yields of 2A–2D must not be summed as independent populations; separate intermediate exclusion and duplicate counts and record-level decisions are not tabulated here. The reported yields are historical, whereas Appendix S1 contains subsequently reconstructed bibliographic search syntax; no later rerun results are reported.

Search component	Database	Records retrieved	Final run	Reconstructed strategy ID
**Core liability search**	PubMed/MEDLINE	452	25 August 2026	Core-PM
**Core liability search**	Scopus	827	25 August 2026	Core-SC
**Core liability search**	Web of Science Core Collection	506	25 August 2026	Core-WOS
**2A Clinical judgement / professional standards**	PubMed/MEDLINE	69	25 August 2026	2A-PM
**2A Clinical judgement / professional standards**	Scopus	187	25 August 2026	2A-SC
**2A Clinical judgement / professional standards**	Web of Science Core Collection	91	25 August 2026	2A-WOS
**2B Autonomy / consent / communication / documentation**	PubMed/MEDLINE	127	25 August 2026	2B-PM
**2B Autonomy / consent / communication / documentation**	Scopus	258	25 August 2026	2B-SC

**2B Autonomy / consent / communication / documentation**	Web of Science Core Collection	147	25 August 2026	2B-WOS
**2C Organisational/system factors / causation / retrospective assessment**	PubMed/MEDLINE	10	25 August 2026	2C-PM
**2C Organisational/system factors / causation / retrospective assessment**	Scopus	17	25 August 2026	2C-SC
**2C Organisational/system factors / causation / retrospective assessment**	Web of Science Core Collection	11	25 August 2026	2C-WOS
**2D Patient safety / defensive medicine / health-system consequences**	PubMed/MEDLINE	106	25 August 2026	2D-PM
**2D Patient safety / defensive medicine / health-system consequences**	Scopus	273	25 August 2026	2D-SC
**2D Patient safety / defensive medicine / health-system consequences**	Web of Science Core Collection	143	25 August 2026	2D-WOS

Search-history reconstruction note

The retained citation exports preserve retrieved records and database-specific yields, but not the literal historical query strings or interface filters. The strings below were developed after the searches to approximate the recorded concept architecture, date range and field conventions. They are not a verbatim search history and cannot independently reproduce the historical results. No results of a later rerun are included here.

Supplementary S3. Reconstructed Database-Specific Search Strategies

The following syntax was reconstructed after completion of the searches. It is an approximate account of the documented concept architecture and database-specific fields, not an authenticated historical strategy. Date limits and interface filters may not be represented exactly; the counts in Table S2 must not be interpreted as results of running these reconstructed strings.

Core-PM. Core liability search — PubMed/MEDLINE

("Obstetrics"[Mesh] OR "Gynecology"[Mesh] OR obstetric*[tiab] OR gynecolog*[tiab] OR gynaecolog*[tiab]) AND ("Malpractice"[Mesh] OR "Liability, Legal"[Mesh] OR malpractice[tiab] OR "medical liability"[tiab] OR "professional liability"[tiab] OR negligenc*[tiab] OR litigation[tiab] OR "liability claim*"[tiab] OR "malpractice claim*"[tiab]) AND ("2015/01/01"[Date - Publication] : "2026/08/25"[Date - Publication])

Core-SC. Core liability search — Scopus

TITLE-ABS-KEY(obstetric* OR gynecolog* OR gynaecolog*) AND TITLE-ABS-KEY(malpractice OR "medical liability" OR "professional liability" OR negligenc* OR litigation OR "liability claim*" OR "malpractice claim*") AND PUBYEAR > 2014 AND PUBYEAR < 2027

Core-WOS. Core liability search — Web of Science Core Collection

TS=(obstetric* OR gynecolog* OR gynaecolog*) AND TS=(malpractice OR "medical liability" OR "professional liability" OR negligenc* OR litigation OR "liability claim*" OR "malpractice claim*") AND PY=(2015-2026)

2A-PM. 2A Clinical judgement / professional standards — PubMed/MEDLINE

("Obstetrics"[Mesh] OR "Gynecology"[Mesh] OR obstetric*[tiab] OR gynecolog*[tiab] OR gynaecolog*[tiab]) AND ("Malpractice"[Mesh] OR "Liability, Legal"[Mesh] OR malpractice[tiab] OR "medical liability"[tiab] OR "professional liability"[tiab] OR negligenc*[tiab] OR litigation[tiab] OR "liability claim*"[tiab] OR "malpractice claim*"[tiab]) AND ("clinical judgment"[tiab] OR "clinical judgement"[tiab] OR "clinical decision-making"[tiab] OR "clinical decision making"[tiab] OR "standard of care"[tiab] OR guideline*[tiab] OR protocol*[tiab] OR "professional standard*"[tiab]) AND ("2015/01/01"[Date - Publication] : "2026/08/25"[Date - Publication])

2A-SC. 2A Clinical judgement / professional standards — Scopus

TITLE-ABS-KEY(obstetric* OR gynecolog* OR gynaecolog*) AND TITLE-ABS-KEY(malpractice OR "medical liability" OR "professional liability" OR negligenc* OR litigation OR "liability claim*" OR "malpractice claim*") AND TITLE-ABS-KEY("clinical judgment" OR "clinical judgement" OR "clinical decision-making" OR "clinical decision making" OR "standard of care" OR guideline* OR protocol* OR "professional standard*") AND PUBYEAR > 2014 AND PUBYEAR < 2027

2A-WOS. 2A Clinical judgement / professional standards — Web of Science Core Collection

TS=(obstetric* OR gynecolog* OR gynaecolog*) AND TS=(malpractice OR "medical liability" OR "professional liability" OR negligenc* OR litigation OR "liability claim*" OR "malpractice claim*") AND TS=("clinical judgment" OR "clinical judgement" OR "clinical decision-making" OR "clinical decision making" OR "standard of care" OR guideline* OR protocol* OR "professional standard*") AND PY=(2015-2026)

2B-PM. 2B Autonomy / consent / communication / documentation — PubMed/MEDLINE

("Obstetrics"[Mesh] OR "Gynecology"[Mesh] OR obstetric*[tiab] OR gynecolog*[tiab] OR gynaecolog*[tiab]) AND ("Malpractice"[Mesh] OR "Liability, Legal"[Mesh] OR malpractice[tiab] OR "medical liability"[tiab] OR "professional liability"[tiab] OR negligenc*[tiab] OR litigation[tiab] OR "liability claim*"[tiab] OR "malpractice claim*"[tiab]) AND ("informed consent"[tiab] OR autonom*[tiab] OR communication[tiab] OR documentat*[tiab] OR "shared decision-making"[tiab] OR "shared decision making"[tiab] OR "patient preference*"[tiab] OR refusal[tiab]) AND ("2015/01/01"[Date - Publication] : "2026/08/25"[Date - Publication])

2B-SC. 2B Autonomy / consent / communication / documentation — Scopus

TITLE-ABS-KEY(obstetric* OR gynecolog* OR gynaecolog*) AND TITLE-ABS-KEY(malpractice OR "medical liability" OR "professional liability" OR negligenc* OR litigation OR "liability claim*" OR "malpractice claim*") AND TITLE-ABS-KEY("informed consent" OR autonom* OR communication OR documentat* OR "shared decision-making" OR "shared decision making" OR "patient preference*" OR refusal) AND PUBYEAR > 2014 AND PUBYEAR < 2027

2B-WOS. 2B Autonomy / consent / communication / documentation — Web of Science Core Collection

TS=(obstetric* OR gynecolog* OR gynaecolog*) AND TS=(malpractice OR "medical liability" OR "professional liability" OR negligenc* OR litigation OR "liability claim*" OR "malpractice claim*") AND TS=("informed consent" OR autonom* OR communication OR documentat* OR "shared decision-making" OR "shared decision making" OR "patient preference*" OR refusal) AND PY=(2015-2026)

2C-PM. 2C Organisational/system factors / causation / retrospective assessment — PubMed/MEDLINE

("Obstetrics"[Mesh] OR "Gynecology"[Mesh] OR obstetric*[tiab] OR gynecolog*[tiab] OR gynaecolog*[tiab]) AND ("Malpractice"[Mesh] OR "Liability, Legal"[Mesh] OR malpractice[tiab] OR "medical liability"[tiab] OR "professional liability"[tiab] OR negligenc*[tiab] OR litigation[tiab] OR "liability claim*"[tiab] OR "malpractice claim*"[tiab]) AND (("organisational factor*"[tiab] OR "organizational factor*"[tiab] OR "system factor*"[tiab] OR "institutional factor*"[tiab] OR staffing[tiab] OR resource*[tiab] OR referral[tiab]) AND (causation[tiab] OR causal*[tiab] OR retrospective[tiab] OR hindsight[tiab])) AND ("2015/01/01"[Date - Publication] : "2026/08/25"[Date - Publication])

2C-SC. 2C Organisational/system factors / causation / retrospective assessment — Scopus

TITLE-ABS-KEY(obstetric* OR gynecolog* OR gynaecolog*) AND TITLE-ABS-KEY(malpractice OR "medical liability" OR "professional liability" OR negligenc* OR litigation OR "liability claim*" OR "malpractice claim*") AND (TITLE-ABS-KEY("organisational factor*" OR "organizational factor*" OR "system factor*" OR "institutional factor*" OR staffing OR resource* OR referral) AND TITLE-ABS-KEY(causation OR causal* OR retrospective OR hindsight)) AND PUBYEAR > 2014 AND PUBYEAR < 2027

2C-WOS. 2C Organisational/system factors / causation / retrospective assessment — Web of Science Core Collection

TS=(obstetric* OR gynecolog* OR gynaecolog*) AND TS=(malpractice OR "medical liability" OR "professional liability" OR negligenc* OR litigation OR "liability claim*" OR "malpractice claim*") AND (TS=("organisational factor*" OR "organizational factor*" OR "system factor*" OR "institutional factor*" OR staffing OR resource* OR referral) AND TS=(causation OR causal* OR retrospective OR hindsight)) AND PY=(2015-2026)

2D-PM. 2D Patient safety / defensive medicine / health-system consequences — PubMed/MEDLINE

("Obstetrics"[Mesh] OR "Gynecology"[Mesh] OR obstetric*[tiab] OR gynecolog*[tiab] OR gynaecolog*[tiab]) AND ("Malpractice"[Mesh] OR "Liability, Legal"[Mesh] OR malpractice[tiab] OR "medical liability"[tiab] OR "professional liability"[tiab] OR negligenc*[tiab] OR litigation[tiab] OR "liability claim*"[tiab] OR "malpractice claim*"[tiab]) AND ("patient safety"[tiab] OR "defensive medicine"[tiab] OR "defensive practice"[tiab] OR "quality of care"[tiab] OR "risk management"[tiab] OR "health system*"[tiab]) AND ("2015/01/01"[Date - Publication] : "2026/08/25"[Date - Publication])

2D-SC. 2D Patient safety / defensive medicine / health-system consequences — Scopus

TITLE-ABS-KEY(obstetric* OR gynecolog* OR gynaecolog*) AND TITLE-ABS-KEY(malpractice OR "medical liability" OR "professional liability" OR negligenc* OR litigation OR "liability claim*" OR "malpractice claim*") AND TITLE-ABS-KEY("patient safety" OR "defensive medicine" OR "defensive practice" OR "quality of care" OR "risk management" OR "health system*") AND PUBYEAR > 2014 AND PUBYEAR < 2027

2D-WOS. 2D Patient safety / defensive medicine / health-system consequences — Web of Science Core Collection

TS=(obstetric* OR gynecolog* OR gynaecolog*) AND TS=(malpractice OR "medical liability" OR "professional liability" OR negligenc* OR litigation OR "liability claim*" OR "malpractice claim*") AND TS=("patient safety" OR "defensive medicine" OR "defensive practice" OR "quality of care" OR "risk management" OR "health system*") AND PY=(2015-2026)

Language restriction audit

The retained Scopus and Web of Science exports contain non-English records, while the retained PubMed thematic export contains English-language records only; the historical PubMed language-filter setting cannot be reconstructed from the missing original database history. During manual selection, the legal specialist did not include publications in languages she could not read. The supplement does not provide a count of exclusions on this basis, and this language restriction limits the breadth of the review.
